# Coping Strategies and Psychopathological Responses Among Medical and Non-medical Professionals – a Cross-Sectional Online Survey

**DOI:** 10.3389/fpsyt.2021.663224

**Published:** 2021-05-20

**Authors:** Marta Ciułkowicz, Julian Maciaszek, Błażej Misiak, Anna Pałȩga, Joanna Rymaszewska, Dorota Maria Szcześniak

**Affiliations:** Department of Psychiatry, Wrocław Medical University, Wroclaw, Poland

**Keywords:** mental health, pandemic, COVID-19, psychopathology, PTSD, coping strategies, healthcare workers

## Abstract

**Background:** The SARS-CoV-2 pandemic was announced on March 11th, 2020, due to a surge of newly confirmed cases that significantly impacted populations worldwide, both directly and indirectly. Based on past epidemics research, the mental health implications of introduced restrictions should be expected and adequately addressed irrespective of the practiced profession.

**Objective:** The study aimed to explore psychopathological responses, including post-traumatic stress disorder (PTSD), concerning coping strategy clusters during the COVID-19 pandemic among medical and non-medical workers.

**Methods:** A cross-sectional web survey of the general population of internet users was performed from March 16th to April 26th, 2020, in Poland during the first peak of COVID-19 cases. A sample of 1,831 professionally active respondents, 64.0% of which pursuing a medical career, filled out General Health Questionnaire-28 (GHQ-28), The Impact of Event Scale-Revised (IES-R), and MiniCOPE, along with the socio-demographic questionnaire exploring personal as well as the work-related possibility of direct exposure to contagion and availability of proper protection, contact with the infected without accurate protective measures as well as the adequacy of workers when compared settings.

**Results:** Individuals labeled with specific clusters had significantly different psychopathological manifestations. Irrespective of performed job maladaptive cluster was associated with significantly higher GHQ-28 and IES-R scores on total subscales and all subscales compared to those representing the non-specific and adaptive cluster. Similar findings were observed concerning the frequency of the GHQ-28 positive score. Moreover, the non-specific cluster was associated with significantly higher GHQ-28 total scores among medical professionals. However, GHQ-28 positive scores were significantly more frequent in medical workers using adaptive clusters when compared to non-specific. Such relations were not observed in the non-medical group.

IES-R total and subscales' scores did not significantly vary within medical and non-medical groups when adaptive and non-specific clusters were compared. Pursuing a non-medical career was found to be a determinant of lower scores, while female sex was observed to be determinant of higher scores in both GHQ-28 and IES-R scales.

**Conclusions:** Positive screening for psychopathological and PTSD symptoms was expected regardless of the analyzed groups' coping strategies. Given the dramatically developing situation of the COVID-19 pandemic, support initiatives grounded in research evidence may be essential for maintaining the mental well-being and resilience of both the medical and non-medical workforce.

## Introduction

In mid-December 2019, the novel coronavirus disease (COVID-19) was described in Wuhan, China, due to the SARS-CoV-2 contagion. A rapid spread of the virus facilitated by globalization resulted in WHO's pandemic declaration on March 11th 2020. As of October 27th, 43 million cases were reported globally, with a mortality rate exceeding one million (1). In spite of the constitution of a newly discovered coronavirus global health emergency shifted scientists' attention to defining clinical picture and developing treatment as well as prophylaxis, so far, accumulated knowledge is rather fragmentary. Although knowledge of emotional responses to the COVID-19 pandemic remains scarce, there is no doubt that the resilience of diverse communities worldwide was considerably challenged both directly and indirectly. Among a wide range of reported manifestations, there are depression, insomnia, anxiety, fear, anger, confusion, or post-traumatic stress symptoms, along with considerable disruptions of everyday routine and stigmatization (2–6). The reported manifestations are not COVID-19 specific.

The outbreak of SARS was regarded as a mental health catastrophe (7). PTSD was the most prevalent long-term psychiatric condition, followed by depressive disorder (8). In a cross-sectional study on a general population, Sim et al. (9), found significant rates of SARS-related psychiatric and post-traumatic comorbidities (22.9 and 25.8%, respectively) 16 weeks after the first local outbreak of SARS in Singapore. They found that psychiatric morbidity was associated with a high level of post-traumatic symptoms and was associated with the increased use of denial and planning as coping measures (9). An online survey addressed to the general population in China at the turn of January and February 2020 showed that most respondents, the majority were women of younger age groups (24–30.8 years of age); described the immediate impact of the Covid-19 epidemic as moderate to severe; more than a quarter declared severe anxiety while 16.5% suffered severe depressive signs (10). In a cross-sectional study in China, demographic data and mental health measurements from 1,257 health care workers were collected. The survey revealed a high prevalence of mental health symptoms among health care workers treating patients with COVID-19. A considerable number of participants had symptoms of depression (634, 50.4%), anxiety (560, 44.6%), insomnia (427, 34.0%), and distress (899, 71.5%). Lai et al. (11) indicated that being a woman and having an intermediate technical title were associated with experiencing severe depression, anxiety, and distress (11). Li et al. (12) also found that although no significant differences were observed between the severity of vicarious traumatization in the non-front-line nurses and the general public, its severity was significantly higher than that of the front-line nurses in close contact with patients with COVID-19 Maciaszek et al. (13) based on a national survey in Poland, aimed to compare psychopathological expressions during the COVID-19 pandemic in medical and non-medical professionals. Out of 2,039 participants, 1,216 (59.6%) individuals represented medical professions while 823 (40.4%) pursued non-medical occupations. Regardless of career, the vast majority of respondents were women (80.0% among medical professionals and 74.4% among non-medical professionals). They found the prevalence of anxiety, insomnia, and somatic symptoms among medical professionals were higher than in non-medical workers. Also, the determinants of psychopathological expressions in these two groups differ in terms of age, care for an elderly or disabled person, contact with COVID-19 at work, and contact with COVID-19 without protection measures (13).

Among the most prominent challenges medical professionals face amid the pandemic are fear of erratic recommendations, working overtime, fear of infection, passing it on family members, using personal protective equipment, and treating fellow workers and critically ill patients (14–16). A consistent pattern of a detrimental impact on the medical staff's psychological and physical well-being was already discussed concerning past outbreaks (14, 17–19). The considerable job-specific hardship put on medical workers during a possible epidemic was emphasized in an Australian study by Martinese et al. (2009) (19). Their study explored work attitudes to two hypothetical influenza scenarios: (a) a single patient admitted with avian influenza; and (b) multiple patients admitted with a new strain of human influenza during a pandemic. The results indicate the majority of respondents, primarily female (two-thirds), aged between 21 and 50 years (three-quarters), and nurses (44%), would not show up for work unless adequate vaccination or antiviral drugs were available at hand undeterred of accessibility of necessary preventive measures. Joob et al. (2020) (20), citing a study by Yasri et al. (2020) (21) underline aggressive attitude toward doctors, such as frequent direct verbal insults or intentional coughing at medical staff in Thailand. As the authors point out, such behavior was not previously observed and might be attributed to the coronavirus outbreak because people are under much stress during a crisis. Additionally, Li et al. (12) highlight the vicarious traumatization of health care workers with no direct exposure to SARS-CoV-2 mirrors levels observed in the general population and consequently affects their everyday work under the extraordinary pressure of the epidemiological crisis (11). Moreover (20), in a study where the majority of participants were between the ages of 18 – 30 years (42.4%) and 31 – 40 years (60.7%), described that working with stressed colleagues provides additional psychological stress in medical respondents irrespective of age (22).

Considering the magnitude of distress and social repercussions of past outbreaks, psychological problems should be expected (23) and appropriately addressed to enhance public health response (24). The great variety of responses and their moderators should be considered mitigating the outbreak's impact. One of the essential factors that may impact individual responses to the outbreak related to stress are coping strategies. To a certain degree, one's performance in the face of stress relates to used coping strategies that may boost not only the health-related quality of life (25) but also impact the prevalence of anxiety and depression (26), insomnia (27), or post-traumatic stress disorder (28). Based on the definition created by Lazarus et al. (29) coping combines both cognitive and behavioral attempts to manage internal and external requirements related to an incredibly stressful event. As Doron et al. (30) point out, many studies investigating the relationships between cognitive coping and mental health adopt a dichotomous coping view. Such a dichotomous approach has strengths and weaknesses. However, as others have identified, instead of focusing on a single coping strategy examining cognitive and behavioral coping strategies in terms of “profiles” that exist across individuals using cluster analytical procedures might be a better approach (30–33). The multidimensional nature of coping has already been used in different contexts (34, 35) and showed that clustering coping actions into categories and thus creating coping strategy clusters reflects actual psychological outcome more adequately as one can use more than one coping strategy as well as combine adaptive and maladaptive strategies at once. In line with the theory proposed by Lazarus and Folkman (29) mentioned above, we wanted to find out the relationship between peoples coping strategies in relation to stressful events such as Covid-19 pandemic. Therefore, the primary aim of this study was to explore the following research questions:

- What are the most prominent combinations of coping strategies in the professionally active group during the pandemic?
- What individual factors and psychopathological symptoms differentiate the coping strategy clusters among the study sample?
- What is the difference in relations between coping strategies clusters and the severity as well as the prevalence of psychopathological symptoms in medical and non-medical groups?
- What are the key determinants of the psychopathological symptoms in the analyzed group?

## Materials and Methods

### Recruitment

Information was collected through the cross-sectional online survey available to the participants between March 16th, 2020, and April 26th, 2020, in Poland. The survey was made available to the public 12 days after confirmation of the first case of the SARS-CoV-2 contagion and included the sudden surge in COVID-19 cases followed by subsequent restrictions to curb the epidemic (36). The snowball sampling method was used to recruit adult representatives of both medical and non-medical professions through social media and email addresses. The survey was posted on social media groups, forums, and websites, both health-related and not. Researchers used their social backgrounds to distribute the survey among their medical and non-medical friends and families to a lesser extent. The healthcare professionals included doctors, nurses, paramedics, and allied medical workers such as pharmacists, physiotherapists, occupational therapists, psychologists, technicians, and administrators. Only wholly filled questionnaires met the inclusion criteria for the analysis. Consent for participation and record of sent data was required. Respondents were informed that the study is entirely voluntary, anonymous and not participating will not negatively affect them. This information was stated in the box before the questionnaire. They were also allowed to stop participating and were assured that the researchers would maintain the records' confidentiality. Submitting a filled questionnaire indicated that respondents had read the study's goal and description, reached adulthood, and agreed to the terms described and participation in the research. The Ethics Committee approved the study protocol at Wroclaw Medical University, Poland (approval number: 188/2020). The study was performed by the principles of the Declaration of Helsinki.

### Measures

#### General Health Questionnaire-28 (GHQ-28)

The prevalence of psychopathological symptoms was measured with a 28-item General Health Questionnaire-28 (GHQ-28) including four subscales: somatic symptoms (items 1, 3, 4, 8, 12, 14, 16), anxiety and insomnia (items 2, 7, 9, 13, 15, 17, 18), social dysfunction (items 5, 10, 11, 25, 26, 27, 28) as well as severe depression (items 6, 19, 20, 21, 22, 23, 24). The scope of possible answers incorporates a 4-point Likert scale (0 – not at all, 1 – no more than usual, 2 – rather more than usual, 3 – much more than usual). The range of possible outcomes extends from 0 to 84, with higher scores indicating higher levels of distress. The cut-off score for clinically relevant symptoms was set at 24 points (37) and thus outcomes exceeding this score were labeled as positive GHQ-28. According to Makowska et al. (38), the GHQ-28 Cronbach's alpha was estimated at 0.93 when the Polish version was used and it was evaluated to be 0.94 in the current sample.

#### MiniCOPE

Coping strategies when approaching threatening situations were measured using a 28-item MiniCOPE questionnaire. Respondents are to choose an answer on a 4-point Likert scale (0 - I hardly ever do that, 1 - I rarely do that, 2 - I often do that, 3 - I almost always do that) best representing their relation to each of 14 subscales that are: active coping (items 2, 7), planning (items 14, 25), positive reframing (items 12, 17), acceptance (items 20, 24), sense of humor (items 18, 28), turning to religion (items 22, 27), seeking emotional support (items 5, 15), seeking instrumental support (items 10, 23), self-distraction (items 1, 19), denial (items 3, 8), venting (items 9, 21), substance use (items 4, 11), behavioral disengagement (items 6, 16) and self-blame (items 13, 26). Possible outcomes in each subscale vary between 0 and 3, with a higher score suggesting more frequent use of specific coping strategies when stressed. The constancy of the test was described satisfactory and split-half reliability for 14 subscales was estimated at 0.86 (39). Cronbach alpha in our study was estimated at 0.76 in our study.

#### The Impact of Event Scale-Revised (IES-R)

Self-reported distress related to traumatic events was measured with The Impact of Event Scale-Revised (IES-R), which is a 22-item questionnaire falling into three subscales: intrusion (items 1, 2, 3, 6, 9, 14, 16, 20), hyperarousal (items 4, 10, 2, 15, 18, 19, 21) along with avoidance (items 5, 7, 8, 11, 13, 17, 22). The choices of the 5-point Likert scale ranges from not at all to definitely yes. There is no fixed cut-off score; however, according to the study by Juczyński et al. (40) on a Polish adaptation of IES-R cut-off score varies from 30 to 33 points. Thus, total outcomes exceeding 30 insinuate developing PTSD symptoms in reaction to trauma. The IES-R Cronbach's alpha was established at 0.92 for the scale in general for the Polish adaptation of the scale (40), and it was equal to 0.94 in the group analyzed in this paper.

#### Socio-Demographic Survey

Additionally, the socio-demographic survey containing questions about socio-demographic data and COVID-19 impact on participants were collected. The socio-demographic questionnaire involved data on general demographic characteristics such as age, sex, place of residence, work settings, being a doctor or a nurse, length of service counted in years, working hours per week, relationship status, having children, and taking care of the disabled or senior person in private life. COVID-19-related questions explored direct exposure to contagion and being provided with proper protection at work, contact with the infected without accurate protective measures, and the adequacy of workers compared to workload. The questions regarding the pandemic explored subjective assessments of the respondents.

### Data Analysis

Statistical analysis was performed using the R (version 3.6) with *psych, car* and *MuMIn* packages. Moreover, in order to perform power analysis for GHQ-28 total score as well as IES-R total score, G*Power (3.1.9.6) Programmer was used. Results of performed analyses were considered significant if the *p*-value was <0.05. The effect size for power analysis was estimated at ~99% for GHQ-28 and 73% for IES-R when medical and non-medical groups were compared.

#### The Most Prominent Combinations of Coping Strategies in the Professionally Active Group During the Pandemic

Cluster analysis (k-means) was performed on the group of 2,038 respondents representing the general population of internet users. In the process of the fit index analysis, an optimal number of three coping strategies clusters emerged. For this study, outcomes of inactive and unemployed people were excluded as only 207 replies were obtained from non-working respondents. Clustered scores of 1,831 professionally active representatives of both medical and non-medical workforce were analyzed. Composition of coping strategies between emerged clusters were compared using the Mann-Whitney U test. Holm method was used to perform *post-hoc* analysis. Additionally, we performed the analysis of covariance (ANCOVA) to control for the effects of sex and age.

#### General and Psychopathological Characteristics With Respect to Clusters of Predominant Coping Strategies

GHQ-28 and IES-R scores, both totals, and particular domains were calculated and complemented with socio-demographic survey covering personal background. Obtained results were compared using Kruskal-Wallis rank sum test (continuous variables) and the chi-square (categorical variables) with all three coping strategy clusters in the general population of internet. Holm correction was used to perform *post-hoc* analysis.

#### Psychopathological Outcomes Regarding Particular Cluster of Coping in Medical and Non-medical Workers

The outcomes of GHQ-28 and IES-R were compared between clustered health care professionals and workers of other professions using the Mann-Whitney U test when continuous variables were considered and the chi-square, including categorical variables. Analysis of covariance (ANCOVA) was performed to control for the effects of sex an age on GHQ-28 and IES-R outcomes. Holm was used in *post-hoc* analysis. Moreover, differences between clusters in medical and non-medical groups in terms of percentage of positive GHQ-28 outcomes were analyzed using a logistic regression model.

#### Key Determinants of Psychopathological Symptoms in the Analyzed Group

To identify the key determinants of psychopathological manifestations measured with GHQ-28 and IES-R in the analyzed group, best subsets regression was performed. Variables which significantly differentiated coping strategy clusters were involved into the models with exception of place of residence, working hours per week that distinguished clusters to a lesser extent (*p* > 0.01). In the next step, variance inflation factor (VIF) was estimated. As age and length of service had VIF exceeding 10, age was removed from the models. Subsequently, obtained models were selected using Bayesian Information Criterion (BIC) criterion and averaged.

## Results

### Participants and Clusters of Predominant Coping Strategies

Participants were 1,173 health care professionals and 658 individuals representing non-medical professions. General characteristics of this sample were reported in our previous publication (13). Our analysis revealed three clusters (Table 1). The first one, referred to as the non-specific cluster, included participants who scored below the mean of all subscales measuring specific coping strategies (a lack of predominant coping strategy). The second cluster labeled maladaptive included respondents who scored above the mean of subscales measuring the likelihood of using denial, venting, substance use, behavioral disengagement, and self-blame. Finally, the adaptive cluster group scored above the mean of subscales measuring the odds of using active coping, planning, positive reframing, seeking emotional and instrumental support. Supplementary Figure 1 presents histograms for the odds of using specific coping strategies at distinct clusters.

**Table 1:** Detailed characteristics of the clusters regarding coping strategies.

**Coping style**	**Cluster 1 Non-specific**	**Cluster 2 Maladaptive**	**Cluster 3 Adaptive**	***Post hoc* comparisons**	**P adj**
	***N* = 511**	***N* = 742**	***N* = 785**		
	**Median (IQR) Mean ± SD**	**Median (IQR) Mean ± SD**	**Median (IQR) Mean ± SD**		
Active coping	2 (1.5–2)	2 (1.5–2)	2.5 1.5 (2, 3)	1 vs. 2^**^	1 vs. 3^**^
	1.77 ± 0.67	1.79 ± 0.5	2.5 ± 0.47	1 vs. 3^**^	2 vs. 3^**^
				2 vs. 3^**^	
Planning	2 (1.5–2)	2 (1.5–2)	2.5 1.5 (2, 3)	1 vs. 3^**^	1 vs. 3^**^
	1.79 ± 0.65	1.84 ± 0.51	2.54 ± 0.45	2 vs. 3^**^	2 vs. 3^**^
Positive reframing	1.5 1.5 (1, 2)	1.5 (1, 2)	2 (2–2.5)	1 vs. 3^**^	1 vs. 3^**^
	1.51 ± 0.72	1.48 ± 0.62	2.16 ± 0.59	2 vs. 3^**^	2 vs. 3^**^
Acceptance	2 (1.5–2)	2 (1.5–2)	2 (2–2.5)	1 vs. 2^**^	1 vs. 3^**^
	1.85 ± 0.66	1.76 ± 0.52	2.31 ± 0.49	1 vs. 3^**^	2 vs. 3^**^
				2 vs. 3^**^	
Sense of humor	1 (0.5–1.5)	1 (0.5–1.5)	1 (0.5–1.5)	1 vs. 3^**^	1 vs. 2^**^
	0.89 ± 0.6	0.95 ± 0.58	1.13 ± 0.61	2 vs. 3^**^	1 vs. 3^**^
					2 vs. 3^**^
Turning to religion	0.5 (0–1)	1 (0–1.88)	1 (0–2)	1 vs. 2^**^	1 vs. 2^**^
	0.71 ± 0.87	0.93 ± 0.93	1.16 ± 1.03	1 vs. 3^**^	1 vs. 3^**^
				2 vs. 3^**^	2 vs. 3^**^
Seeking emotional support	1 (0.5–1.5)	2 (1.5–2)	2 (2–2.5)	1 vs. 2^**^	1 vs. 2^**^
	1.03 ± 0.64	1.77 ± 0.64	2.15 ± 0.59	1 vs. 3^**^	1 vs. 3^**^
				2 vs. 3^**^	2 vs. 3^**^
Seeking instrumental support	1 (0.5–1.25)	2 (1.5–2)	2 (2–2.5)	1 vs. 2^**^	1 vs. 2^**^
	0.9 ± 0.56	1.81 ± 0.61	2.05 ± 0.59	1 vs. 3^**^	1 vs. 3^**^
				2 vs. 3^**^	2 vs. 3^**^
Self-distraction	1 (0.75–1.5)	2 (1.5–2)	2 (1.5–2)	1 vs. 2^**^	1 vs. 2^**^
	1.23 ± 0.65	1.81 ± 0.56	1.81 ± 0.66	1 vs. 3^**^	1 vs. 3^**^
				2 vs. 3^*^	
Denial	0.5 (0–1)	1 (0.5–1.5)	0.5 (0–1)	1 vs. 2^**^	1 vs. 2^**^
	0.49 ± 0.54	1.08 ± 0.6	0.53 ± 0.57	2 vs. 3^**^	2 vs. 3^**^
Venting	1 (0.5–1.5)	1.5 (1.5–2)	1.5 (1, 2)	1 vs. 2^**^	1 vs.2^**^
	0.97 ± 0.53	1.75 ± 0.48	1.58 ± 0.56	1 vs. 3^**^	1 vs. 3^**^
				2 vs. 3^**^	2 vs. 3^**^
Substance use	0 (0–1)	1 (0–1.5)	0 (0–1)	1 vs. 2^**^	1 vs. 2^**^
	0.4 ± 0.6	0.86 ± 0.83	0.42 ± 0.63	2 vs. 3^**^	2 vs. 3^**^
Behavioral disengagement	0.5 (0–1)	1 (1–1.5)	0.5 (0–0.5)	1 vs. 2^**^	1 vs.2^**^
	0.62 ± 0.55	1.19 ± 0.52	0.37 ± 0.41	1 vs. 3^**^	1 vs. 3^**^
				2 vs. 3^**^	2 vs. 3^**^
Self-blame	1 (0.5–1.5)	2 (1.5–2.5)	1 (0.5–1.5)	1 vs. 2^**^	1 vs. 2^**^
	0.99 ± 0.79	1.81 ± 0.72	0.99 ± 0.66	2 vs. 3^**^	2 vs. 3^**^

*P adj – p-value adjusted for sex and age (ANCOVA). Post-hoc comparison was performed using Holm method. Data expressed as median and interquartile range (IQR) as well as mean and standard deviation (SD)*.

* *p < 0.05*,

** *p < 0.001*.

#### General and Psychopathological Characteristics With Respect to Cluster of Predominant Coping Strategies

There were significant differences between individuals representing specific clusters of predominant coping strategies concerning age, sex, place of residence, work profession (medical vs. non-medical professionals), length of service, working hours per week, having children, and self-perception of protection accuracy at work (Table 2). Individuals representing specific clusters differed significantly in terms of psychopathological manifestation. Respondents from the maladaptive cluster had significantly higher GHQ-28 scores (total score as well as scores of somatic symptoms, anxiety, and insomnia, social dysfunction, and severe depression) and IES-R scores (total score as well as scores of intrusion, arousal, and avoidance) compared to those representing the “non-specific” cluster and the adaptive cluster. Similar findings were observed with respect to the frequency of the GHQ-28 positive score. Also, individuals from the non-specific cluster had significantly higher GHQ-28 scores (total score as well as the score of social dysfunction and severe depression) compared to respondents from the adaptive cluster. In turn, the IES-R score of avoidance was significantly higher in subjects representing the adaptive cluster than in those from the non-specific cluster.

**Table 2:** Descriptive analysis of the clusters regarding all respondents.

		**Cluster 1 Non-specific**	**Cluster 2 Maladaptive**	**Cluster 3 Adpative**	***Post hoc* comparisons**
		***N* = 467**	***N* = 655**	***N* = 709**	
Age		42 (32–51)	34 (28–47)	40 (31–50)	1 >2^**^
		43.04 ± 12.76	37.79 ± 11.56	41.09 ± 11.64	1 > 3^*^ 2 <3^**^
Sex	Female	297 (21.0%%)	564 (40.0%)	552 (39.0%)	1 vs. 2^**^ 1 vs. 3^**^
	Male	170 (40.7%)	91 (21.8%)	157 (37.6%)	2 vs. 3^**^
Place of residence	Urban	433 (24.8%)	631 (36.2%)	680 (39.0%)	1 vs. 2^*^ 1 vs. 3^*^
	Countryside	34 (39.1%)	24 (27.6%)	29 (33.3%)	
Work profession	Medical	268 (22.8%)	452 (38.6%)	453 (38.6%)	1 <2^**^
	Non-medical	199 (30.2%)	203 (30.9%)	256 (38.9%)	1 <2^*^
Length of service (years)		18 (7–27)	10 (3–23)	15 (6–25)	1 > 2^*^
		18.73 ± 12.98	13.58 ± 11.8	16.57 ± 11.88	1 > 3^*^
					2 <3^*^
Working hours per week		40 (40–50)	40 (40–50)	40 (37–48)	1 vs. 3^*^
		43.51 ± 13.95	43.65 ± 13.52	41.98 ± 13.18	2 vs. 3^*^
Being in relationship	Yes	363 (25.8%)	488 (34.7%)	556 (39.5%)	
	No	104 (24.5%)	167 (39.4%)	153 (36.1%)	
Having children	Yes	294 (29.0%)	308 (30.4%)	412 (40.6%)	1 vs. 2^**^
					2 vs. 3^**^
	No	173 (21.2%)	347 (42.4%)	297 (36.4%)	
Taking care of disabled or senior person in private life	Yes	80 (26.1%)	107 (35.0%)	119 (38.9%)	
	No	385 (25.3%)	547 (36.0%)	588 (38.7%)	
Direct contact with the infected at work	Yes	84 (28.7%)	105 (35.8%)	104 (35.5%)	
	No	382 (24.9%)	549 (35.7%)	605 (39.4%)	
Accurate protectionat work	Yes	244 (28.0%)	245 (28.2%)	381 (43.8%)	1 vs. 2^*^ 2 vs. 3^*^
	No	223 (23.2%)	409 (42.6%)	328 (34.2%)	
Contact with (possibly) infected without accurate protection	Yes	54 (22.3%)	85 (35.1%)	103 (42.6%)	
	No	413 (26.0%)	570 (35.9%)	606 (38.1%)	
Number (adequacy) of workers when compared to workload	Too few	244 (24.0%)	382 (37.6%)	389 (38.4%)	
	Other responses	222 (27.3%)	272 (33.5%)	318 (39.2%)	
GHQ-28	Total	22 (14–33)	35 (24–45.5)	21 (14–29)	1 <2^**^
		25.28 ± 14.75	35.81 ± 15.11	22.88 ± 11.75	1 > 3^*^
					2 > 3^**^
	Positive	216 (21.4%)	499 (49.4%)	295 (29.2%)	1 <2^**^
					2 > 3^**^
	Somatic symptoms	5 (3–9)	9 (5.5–13)	5 (3–8)	1 <2^**^
		6.42 ± 4.45	9.24 ± 4.74	6.08 ± 4.08	2 > 3^**^
	Anxiety and insomnia	7 (3–12)	12 (8–16)	7 (4–11)	1 <2^**^
		7.95 ± 5.51	11.84 ± 5.17	7.88 ± 4.99	2 > 3^**^
	Social dysfunction	7 (6–10)	9 (7–12)	7 (6–8)	1 <2^**^
		8.02 ± 3.23	9.66 ± 3.64	7.1 ± 2.87	1 > 3^**^
					2 > 3^**^
	Severe depression	2 (0–4)	4 (2–7)	1 (0–2)	1 <2^**^
		2.89 ± 3.78	5.07 ± 4.35	1.81 ± 2.49	1 > 3^**^
					2 > 3^**^
IES-R	Total	29 (18–42)	45 (36–55)	31 (19–44)	1 <2^**^
		30.29 ± 16.84	44.37 ± 15.55	32.05 ± 16.2	2 > 3^**^
	Intrusion	1.25 (0.62–2)	2.12 (1.5–2.75)	1.38 (0.75–2.12)	1 <2^**^
		1.37 ± 0.93	2.11 ± 0.86	1.44 ± 0.87	2 > 3^**^
	Arousal	1.29 (0.71–2)	2.14 (1.57–2.71)	1.29 (0.86–2)	1 <2^**^
		1.39 ± 0.88	2.09 ± 0.85	1.43 ± 0.82	2 > 3^**^
	Avoidance	1.43 (0.86–1.86)	1.86 (1.43–2.29)	1.57 (1–2)	1 <2^**^
		1.37 ± 0.74	1.83 ± 0.71	1.5 ± 0.76	1 <3^*^
					2 > 3^**^

*Post hoc comparison was performed using Holm method. Data expressed as median and interquartile range (IQR) as well as mean ± standard deviation (SD) or n (%)*.

* *p < 0.05*,

** *p < 0.001*.

#### Psychopathological Outcomes Regarding a Particular Cluster of Coping in Medical and Non-medical Workers

The levels of psychopathological symptoms concerning clusters of predominant coping strategies in medical and non-medical professionals are in Table 3. Among medical professionals, participants from the maladaptive cluster had significantly higher GHQ-28 scores (total score and scores of all specific subscales) and IES-R scores (total score and scores of all specific subscales) compared to those from the non-specific cluster and the adaptive cluster (after post hoc tests as well as adjustment of age and sex). Similar differences were found for the frequency of the GHQ-28 positive score. Additionally, medical professionals from the adaptive cluster scored significantly lower in the GHQ-28 in total and in somatic symptoms, social dysfunction, and severe depression subscales compared to the non-specific cluster. Intriguingly, the frequency of GHQ-28 positive scores was significantly higher in medical professionals clustered as adaptive when compared to those from the non-specific cluster (after post hoc tests and adjustment of age and sex).

**Table 3:** Comparison of GHQ-28 and IES-R outcomes regarding particular cluster of coping in medical and non-medical workers.

			**Medical**	**P adj**	**Non- medical**	**P adj**
			***N* = 1,173**		***N* = 658**	
			**Median (IQR)**		**Median (IQR)**	
			**Mean ± SD**		**Mean ± SD**	
GHQ-28	Total	Cluster 1	25 (16–35) 26.7 ± 14.4	1 vs. 2 ^**^ 1 vs. 3 ^**^ 2 vs. 3 ^**^	18 (12–31) 23.4 ± 15	1 vs. 2 ^**^ 2 vs. 3 ^**^
		Cluster 2	36 (26–47) 37.1 ± 14.6		30 (21.5–43) 33 ± 15.9	
		Cluster 3	22 (15–30) 23.8 ± 11.8		19 (12.75–27) 21.3 ± 11.6	
	Positive	Cluster 1	141 (19.9%)	1 <2 ^**^ 1 <3 ^*^ 2 > 3 ^**^	75 (25.0%)	2 > 1 ^**^ 3 <2 ^**^
		Cluster 2	363 (51.1%)		136 (45.3%)	
		Cluster 3	206 (29.07%)		89 (29.7%)	
	Somatic symptoms	Cluster 1	6 (3–10) 6.8 ± 4.4	1 vs. 2 ^**^ 1 vs. 3 ^*^ 2 vs. 3 ^**^	5 (2–8) 5.9 ± 4.4	1 vs. 2 ^**^ 2 vs. 3 ^**^
		Cluster 2	9 (6–13) 9.6 (4.6)		8 (4.5–11.5) 8.4 ± 4.9	
		Cluster 3	6 (3–9) 6.4 ± 4.1		5 (2–8) 5.4 ± 3.9	
	Anxiety and insomnia	Cluster 1	8 (4–13) 8.7 ± 5.5	1 vs. 2 ^**^ 2 vs. 3 ^**^	6 (3–10) 7 ± 5.4	1 vs. 2 ^**^ 2 vs. 3 ^**^
		Cluster 2	13 (9–16) 12.4 ± 4.9		10 (6–15) 10.6 ± 5.5	
		Cluster 3	8 (4–12) 8.3 ± 5		7 (3–10) 7.2 ± 4.9	
	Social dysfunction	Cluster 1	7.5 (6–10) 8.3 ± 3.1	1 vs. 2 ^**^ 1 vs. 3 ^**^ 2 vs. 3 ^**^	7 (6–9) 7.7 ± 3.3	1 vs. 2 ^**^ 1 vs. 3 ^*^ 2 vs. 3 ^**^
		Cluster 2	9 (7–12) 9.8 ± 3.6		9 (7–12) 9.4 ± 3.7	
		Cluster 3	7 (6–8) 7.1 ± 2.9		7 (6–8) 7.1 ± 2.8	
	Severe depression	Cluster 1	2 (0–4) 2.9 ± 3.7	1 vs. 2 ^**^ 1 vs. 3 ^**^ 2 vs. 3 ^**^	1 (0–4) 2.8 ± 3.9	1 vs. 2 ^**^ 1 vs. 3 ^**^ 2 vs. 3 ^**^
		Cluster 2	4 (2–8) 5.3 ± 4.3		3 (1–6) 4.6 ± 4.4	
		Cluster 3	1 (0–2) 1.9 ± 2.6		1 (0–2) 1.6 ± 2.3	
IES–R	Total	Cluster 1	30 (18–42) 30.7 ± 16.9	1 vs. 2 ^**^ 2 vs. 3 ^**^	29 (18.25–41) 29.8 ± 16.8	1 vs. 2 ^**^ 2 vs. 3 ^**^
		Cluster 2	45 (35–55) 44.5 ± 15.6		45 (36–55) 44.1 ± 15.5	
		Cluster 3	31 (20–45) 32.8 ± 16		30 (9–42) 30.8 ± 16.6	
	Intrusion	Cluster 1	1.38 (0.62–2) 1.4 ± 0.9	1 vs. 2 ^**^ 2 vs. 3 ^**^	1.25 (0.62–1.94) 1.4 ± 0.9	1 vs. 2 ^**^ 2 vs. 3 ^**^
		Cluster 2	2.12 (1.5–2.75) 2.1 ± 0.9		2.12 (1.56–2.62) 2.1 ± 0.9	
		Cluster 3	1.38 (0.75–2.12) 1.5 ± 0.9		1.25 (0.75–2) 1.4 ± 0.9	
	Arousal	Cluster 1	1.29 (0.71–2) 1.4 ± 0.9	1 vs. 2 ^**^ 2 vs. 3 ^**^	1.29 (0.71–1.86) 1.4 (0.9)	1 vs. 2 ^**^ 2 vs. 3 ^**^
		Cluster 2	2.14 (1.57–2.71) 2.1 ± 0.8		2.14 (1.57–2.71) 2.1 ± 0.9	
		Cluster 3	1.43 (0.86–2) 1.5 ± 0.8		1.29 (0.71–1.86) 1.4 ± 0.8	
	Avoidance	Cluster 1	1.36 (0.86–1.86) 1.3 ± 0.7	1 vs. 2 ^**^ 2 vs. 3 ^**^	1.43 (0.86–2) 1.4 ± 0.8	1 vs. 2 ^**^ 2 vs. 3 ^**^
		Cluster 2	1.86 (1.43–2.29) 1.8 ± 0.7		2 (1.43–2.29) 1.9 ± 0.7	
		Cluster 3	1.57 (1, 2) 1.5 ± 0.7		1.43 (0.96–2) 1.5 ± 0.8	

*P adj – p-value adjusted for sex and age (ANCOVA). Post-hoc comparison was performed using Holm method. Data expressed as median and interquartile range (IQR) as well as mean and standard deviation (SD)*.

* *p < 0.05*,

** *p < 0.001*.

In non-medical professionals, significantly higher scores of GHQ-28 (total score and scores of all specific subscales) and IES-R scores (total score and scores of all specific subscales) were observed in the maladaptive cluster when compared to adaptive and non-specific. Likewise, the frequency of GHQ-28 positive scores was significantly higher when compared to adaptive and non-specific clusters. Moreover, non-medical workers clustered as adaptive scored considerably lower in social dysfunction and severe depression subscales of GHQ-28 when compared to non-specific cluster.

Furthermore, no significant differences were found between IES-R scores (total score and scores of all specific subscales) when adaptive and non-specific clusters were compared within both medical and non-medical groups. Supplementary Figures 2, 3 present histograms of the GHQ-28 and the IES-R scores in medical and non-medical professionals.

#### Key Determinants Regarding Psychopathological Symptoms in the Analyzed Group

Due to varied psychopathological manifestations in given clusters, best subsets regression analysis was performed (Table 4) to explore key determinants of GHQ-28 and IES-R scores in all respondents. Female sex was observed to be the only significant socio-demographic variable defined as a determinant of higher total and all subscales' scores of GHQ-28 and IES-R. Following a non-medical career, in turn, was found to be the only socio-demographic variable associated with less psychopathological symptoms measured with GHQ-28 total score and its' specific subscales of somatic symptoms well as anxiety and insomnia. Having children significantly determined lower scores of only severe depression subscale of GHQ-28.

**Table 4:** Determinants of GHQ-28 and IES-R scores in analyzed group.

	**GHQ-28**					**IES-R**				
	**Predictor**	**Total**	**Somatic symptoms**	**Anxiety and insomnia**	**Social dysfunction**	**Severe depression**	**Total**	**Intrusion**	**Arousal**	**Avoidance**
Female sex	Coeff	6.14^**^	2.14^**^	2.07^**^	1.02^**^	0.77^**^	4.57^**^	2.16^**^	1.82^**^	0.67^*^
	RVI	1.00	1.00	1.00	1.00	1.00	1.00	1.00	1.00	0.21
	95%CI	4.75–7.54	1.68–2.60	1.49–2.65	0.68–1.36	0.41–1.13	2.88–6.25	1.39–2.92	1.19–2.46	−0.45–0.74
Having children	Coeff					−0.39^*^				
	RVI					0.38				
	95%CI					−0.56–0.27				
Non-medical profession	Coeff	−2.11^**^	−0.75^**^	−1.13^**^						
	RVI	1.00	1.00	1.00						
	95%CI	−3.27–0.95	−1.14–0.36	−1.58–0.68						
Length of service	Coeff									
	RVI									
	95%CI									
Accurate protection at work	Coeff									
	RVI									
	95%CI									
Active coping	Coeff							0.42^*^		
	RVI							1.00		
	95%CI							−0.25–0.38		
Planning	Coeff									
	RVI									
	95%CI									
Positive reframing	Coeff	−2.26^**^	−0.58^**^	−0.77^**^	−0.49^**^	−0.44^**^	−1.10^**^	−0.72^**^	−0.57^**^	0.22^*^
	RVI	1.00	1.00	1.00	1.00	1.00	1.00	1.00	1.00	0.29
	95%CI	−2.73–1.80	−0.73–0.43	−0.95–0.58	−0.61–0.37	−0.55–0.33	−1.64–0.55	−0.97–0.46	−0.77–0.36	−0.15–0.28
Acceptance	Coeff				−0.17^*^					
	RVI				0.35					
	95%CI				−0.24–0.12					
Sense of humor	Coeff	−0.62^*^		−0.31^**^			−0.78^*^	−0.38^*^	−0.28^*^	
	RVI	0.22		0.78			0.25	0.37	0.13	
	95%CI	−0.69–0.42		−0.55–0.06			−0.93–0.53	−0.54–0.26	−0.24–0.17	
Turning to religion	Coeff	0.37^*^	0.12^*^	0.19^*^			0.99^**^	0.40^**^	0.33^**^	0.28^**^
	RVI	0.26	0.14	0.92			1.00	1.00	1.00	1.00
	95%CI	−0.26–0.45	−0.07–0.11	0.03–0.32			0.64–1.34	0.25–0.56	0.20–0.46	0.16–0.40
Seeking emotional support	Coeff	−0.90^**^	−0.19^*^	−0.21^*^	−0.18^**^	−0.29^**^	−1.16^**^	−0.33^*^	−0.40^**^	−0.38^**^
	RVI	1.00	0.55	0.43	1.00	1.00	1.00	0.88	1.00	1.00
	95%CI	−1.30–0.50	−0.31–0.10	−0.32–0.14	−0.28–0.09	−0.39–0.19	−1.64–0.68	−0.58–0.00	−0.57–0.22	−0.54–0.23
Seeking instrumental support	Coeff									
	RVI									
	95%CI									
Self-distraction	Coeff			0.25^*^			1.15^**^	0.32^*^	0.29^*^	0.61^**^
	RVI			0.45			1.00	0.27	0.59	1.00
	95%CI			−0.16–0.39			0.60–1.70	−0.22–0.39	−015–0.50	0.43–0.80
Denial	Coeff	1.48^**^	0.45^**^	0.58^**^	0.20^*^	0.29^**^	2.64^**^	0.93^**^	0.74^**^	1.02^**^
	RVI	1.00	1.00	1.00	1.00	1.00	1.00	1.00	1.00	1.00
	95%CI	0.99–1.96	0.28–0.61	0.39–0.76	0.08–0.32	0.17–0.42	2.05–3.24	0.68–1.18	0.51–0.96	0.83–1.20
Venting	Coeff	1.32^**^	0.39^**^	0.59^**^			1.78^**^	0.74^**^	0.77^**^	0.38^**^
	RVI	1.00	1.00	1.00			1.00	1.00	1.00	1.00
	95%CI	0.80–1.84	0.21–0.57	0.37–0.81			1.14–2.42	0.45–1.03	0.52–1.01	0.17–0.59
Substance use	Coeff	1.94^**^	0.57^**^	0.68^**^	0.28^**^	0.46^**^	2.02^**^	0.90^**^	0.80^**^	0.30^**^
	RVI	1.00	1.00	1.00	1.00	1.00	1.00	1.00	1.00	1.00
	95%CI	1.55–234	0.44–0.70	0.53–0.83		0.36–0.57	1.54–2.49	0.69–1.11	0.62–0.97	0.15–0.46
Behavioral disengagement	Coeff	1.81^**^	0.46^**^	0.35^*^	0.47^**^	0.58^**^	0.86^*^	0.48^*^	0.36^*^	
	RVI	1.00	1.00	1.00	1.00	1.00	0.28	0.15	0.68	
	95%CI	1.26–2.36	0.26–0.66	0.14–0.57	0.33–0.61	0.44–0.73	−0.59–1.08	−0.28–0.43	−0.14–0.63	
Self-blame	Coeff	1.30^**^	0.20^*^	0.37^**^	0.24^**^	0.51^**^	2.60^**^	1.26^**^	0.85^**^	0.46^**^
	RVI	1.00	0.73	1.00	1.00	1.00	1.00	1.00	1.00	1.00
	95%CI	0.90–1.69	−0.06–0.36	0.21–0.52	0.14–0.34	0.41–0.62	2.12–3.09	1.04–1.47	0.66–1.04	0.31–0.61

*Results of best subset models regression analysis. Coeff - coefficient, RVI, relative variable importance; 95% CI, 95% confidence interval*.

* *p < 0.05*,

** *p < 0.001*.

In contrast, numerous significant relationships were detected between using specific coping strategies and GHQ-28 and IES-R scores. According to the regression models, strategies such as self-blame, behavioral disengagement, substance use, and denial were determinants of significantly higher outcomes in GHQ-28 and IES-R outcomes (total scores and all subscales' scores). Positive reframing, along with seeking emotional support used as a coping strategy, reduced GHQ-28 and IES-R scores (total scores as well as of all its subscales). Moreover, no significant relations were found between seeking instrumental support, planning, and both scales' outcomes. Using acceptance as a coping strategy significantly determined lower scores of only the social dysfunction subscale of GHQ-28.

Additionally, venting, self-distraction, and turning to religion predicted worse outcomes in total and all IES-R subscales. Furthermore, active coping was significantly related to higher scores in the intrusion subscale of IES-R. The best models accounted for 37% and 33% of the variance of the psychopathological manifestations measured by GHQ-28 and IES-R, respectively.

## Discussion

This study aimed to analyze the most prominent combinations of coping strategies observed in the professionally active study sample during the pandemic. Moreover, it focuses on crucial individual factors and coping strategies that differentiate the severity and occurrence of psychopathological symptoms in the study sample of both medical and non-medical professionals.

A great deal of research has already emphasized cluster analysis legitimacy exploring coping strategy clusters (11, 39–45). It may mirror actual responses to stressful transactions more adequately and help predict possible severe psychological distress and psychopathological symptoms. In our study, three clusters of coping strategies emerged and were tagged as non-specific, maladaptive, and adaptive, taking into account the used combination's prevailing character. Interestingly, no statistically significant differences were found in using strategies of active coping, planning, positive reframing, and acceptance and behavioral disengagement when non-specific and maladaptive strategy clusters were compared. Similarly, such relation was observed regarding denial, substance use, and self-blame in non-specific and adaptive. This suggests that using a single coping strategy may not determine the whole response as adaptive or maladaptive. Umucu et al. (43), examining a sample of individuals with self-reported chronic disorders and disabilities during the COVID-19 pandemic, analyzed single coping strategies and concluded that strategies such as self-distraction, denial, substance use, behavioral disengagement, venting, planning, religion, and self-blame were positively correlated with perceived coronavirus-related stress (43).

According to our research, all respondents using maladaptive coping strategies cluster have more psychopathological symptoms than those clustered as non-specific and adaptive. In turn, belonging to the non-specific cluster implies a greater severity of depressive symptoms and social dysfunction when compared to the adaptive cluster. Similarly, the highest total scores of GHQ-28 and thus, the most significant risk of developing psychopathological symptoms were found in both medical and non-medical groups using coping strategies clustered as maladaptive. Intriguingly, the proportion of positive GHQ-28 observed in medical professionals using strategies clustered as adaptive was significantly higher than those using coping strategies classified as non-specific. Such a relation was not observed among non-medical workers. The job-specific burden of the COVID-19 pandemic on medical workers' mental health has already been widely discussed (22–24, 46, 47).

Novel viral infections pose a unique challenge because of the transmission speed and considerable concentration of infected patients in health care facilities. According to previous studies, healthcare workers reported high-stress levels during the past epidemics (SARS and MERS) (48, 49). This stands in line with our research as a non-medical career was found to be a determinant of better mental health outcomes.

Moreover, our study indicates that people with the most extended work history and long working hours per week used non-specific coping strategies. In comparison, those presenting adaptive coping strategies worked the shortest hours. Furthermore, although working hours from a weekly perspective vary slightly, even a few hours per week could make a difference in developing psychopathological symptoms. Intriguingly, according to our research, not being provided with accurate Personal Protective Equipment had a more noticeable impact on using maladaptive coping strategies than actual direct exposure to contagion. This observation resonates with Szczesniak et al. (46) and Tan et al. (47), where the beneficial impact of protective measures on mental health was emphasized.

The age of the respondents also influences the use of different coping strategies. Different types of stressors are encountered as individuals age, and these differences in stressors, as well as associated life events, have an impact on coping strategies and health outcomes. Although most young adults are at low risk of physical health complications from COVID-19, they may be concerned with secondary consequences. Justo-Alonso et al. (44) showed that young people are more likely to be psychologically affected by COVID-19 significantly, while the oldest showed better psychological responses in general. However, there were slight differences between age groups in our study and adapted coping strategies as respondents were from the same age category. This being said, juxtaposing our results with recent research, psychological response to COVID-19 could vary among different age groups that may have clinical implications such as anxiety and depression.

Additionally, according to Cai et al., the biggest concern of medical workers aged 31–40 years old was passing on viral infection to their young children and parents (22). In our study, such a dilemma was reflected in the more frequent use of maladaptive coping strategies in being a parent. However, this did not apply to being a caregiver of a senior or disabled person privately. Interestingly, when regression analysis of factors influencing worse mental health outcomes was performed, female sex emerged to be the only socio-demographic independent variable predicting significantly worse mental health outcomes. In this context, it might be related to cultural demand to adopt the primary care provider's role.

Post-traumatic stress syndrome has already been described concerning past outbreaks such as SARS (50), Ebola (51) as well as H1N1 (52). Prevalence of COVID-related PTSD amounts between almost 5% (14) to 29%, depending on the used diagnostic tool and cut-off score. The longitudinal research on COVID-19 influence on the general population carried by Wang et al. showed that both initial examination at the start of the epidemic and its follow-up showed IES-R scores exceeding 24 points that suggest the presence of PTSD symptoms. Importantly, no significant reduction was observed in the 1 month (10). According to Lai et al., low distress tolerance facilitated PTSD symptoms, while resilience was not proved to be a preventive factor. In our research, all respondents using coping strategies clustered as maladaptive scored significantly higher in total and all in subscales of IES-R compared to adaptive and non-specific.

Interestingly, respondents using coping strategies clustered as adaptive scored significantly higher in the avoidance subscale of IES-R when compared to the non-specific cluster. This suggests that they may try to get rid of thoughts and emotions resembling the trauma and avoid discussing them. In turn, it may be problematic in the context of establishing a therapeutic relationship with that group or designing proper supporting actions. Furthermore, active coping, which is considered to be an adaptive coping strategy and is associated with taking actions to improve the specific situation, was related to higher intrusion scores and thus recurrent thought and nightmares concerning trauma. This may be connected to lacking expertise and significant restrictions introduced due to the pandemic and frustration and helplessness. Likewise, planning, usually classified as an adaptive strategy, did not significantly determine IES-R scores. This stands in line with the research by Sim et al. (9) regarding experience from the SARS outbreak. Moreover, in our study, only seeking emotional support was found to reduce both totals and all subscales' scores of IES-R and thus mitigate overall discomfort along with specific dimensions of PTSD in contrast to seeking instrumental support even though they are both components of social coping.

The study provides another aspect worth addressing in further research: the pandemic's psychological impact on medical professionals. Recognition of coping strategies used by medical professionals and how the pandemic will influence them is crucial not only at an individual level but also on a collective one. Supporting the mental health of medical staff is a critical part of the public health response. Unless medical services take active measures and adopt a proactive approach, the pandemic's psychological consequences on healthcare staff could be dramatic. Given that the COVID-19 can lead to various mental health outcomes, understanding risk and resilience factors might hold a grand promise for developing specific interventions that aim to restore psychological well-being. Medical professionals armed with holistic training could better identify a fellow front-line worker experiencing anxiety or depression symptoms and provide support while maintaining the fluidity of healthcare in the face of a crisis (pandemic) (53). Buselli et al. (54) highlight in their study the negative impact of secondary trauma on medical professional's mental health in terms of anxiety. They emphasize that the stressful event itself is not so much a risk factor, but the event's perception as traumatic.

### Limitations

Despite its contributions, the present study is not exempt from certain limitations. Our sample might have low representativeness as we did not record the number of individuals approached for participation. The character of the survey was voluntary, self-reported, which may have facilitated selection bias.

Moreover, the sample was not equally distributed for gender as the majority of respondents were women, which may have affected our results. Similarly, data concerning detailed occupations of non-medical respondents was obtained but minding a considerable level of generality and restricted number of received replies; they could not be adequately classified and analyzed from the perspective of operating mode, possible contagion risk, and their consequences for mental health. We also did not inquire about co-occurring mental disorders. Furthermore, the online distribution could have facilitated the participation of those confident with internet use. Likewise, this study's cross-sectional design prohibits statements of causality, and the anonymity of the respondents prevents us from tracking ones needing psychological support. However, providing the workforce with such support demands exploring observed relationships between coping strategies and the severity of psychopathological symptoms in further research.

## Conclusions and Practical Implications

Our findings suggest some reflections about the relations between coping strategies and the severity of psychopathological symptoms among medical and non-medical professionals. One possible determinant of the immediate response to the pandemic outbreak could be employer support. Occupational and behavioral health interventions such as education, health promotion, and anti-stigma interventions directed at those with the most extended work history and long working hours per week who use non-specific coping strategies could prevent them from developing clinically significant psychopathological symptoms. Working schedules should be created thoughtfully to prevent workers from overworking, and proper protection at work should be provided. Moreover, interventions aiming to identify and address one's emotions, such as psychological counseling, may be beneficial regardless of the career.

In contrast, instrumental support may not result in better mental health outcomes. Remarkably, in our research, non-medical background relates to better mental health outcomes, while being a female is associated with developing more psychopathological outcomes. This being said, special attention should be paid to supporting female medical workers. Given the coronavirus pandemic's dynamically developing situation, generating evidence-driven supportive initiatives, respecting inter-subject variability is of the utmost importance in restoring the general public and medical, mental well-being, and resilience workers.

## Data Availability Statement

The raw data supporting the conclusions of this article will be made available by the authors, without undue reservation.

## Author Contributions

MC, JM, and BM designed the research project, the main conceptual ideas and proof outline, conducted the study, interpreted the results, complied the literature sources, wrote the manuscript, and checked the references. AP complied the literature sources, wrote the manuscript, and checked the references. JR and DS conceptualized and designed the study, helped in the interpretation of data, and checked references. All authors contributed to the article and approved the submitted version.

## Conflict of Interest

The authors declare that the research was conducted in the absence of any commercial or financial relationships that could be construed as a potential conflict of interest.
