# Supplementary material for: Coping Strategies and Psychopathological Responses Among Medical and Non-medical Professionals – a Cross-Sectional Online Survey
Source: Front Psychiatry. 2021 May 20;12:663224. doi: 10.3389/fpsyt.2021.663224 (PMC8173082; doi:10.3389/fpsyt.2021.663224)

**Supplementary File**

Histograms for the odds of using specific coping strategies at distinct clusters.


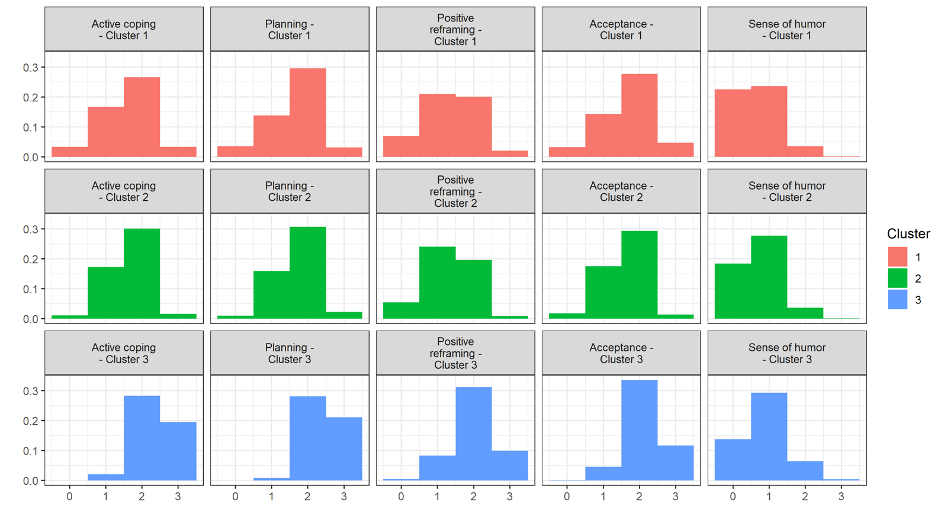


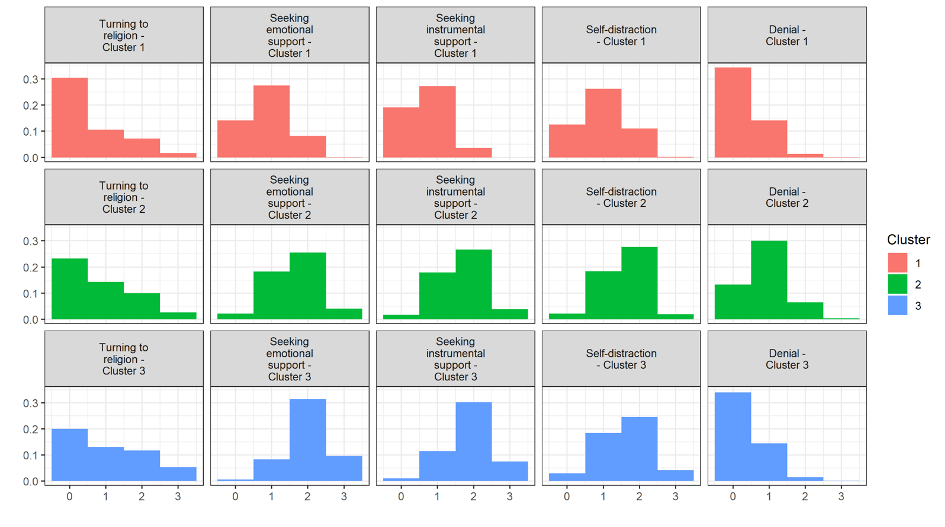


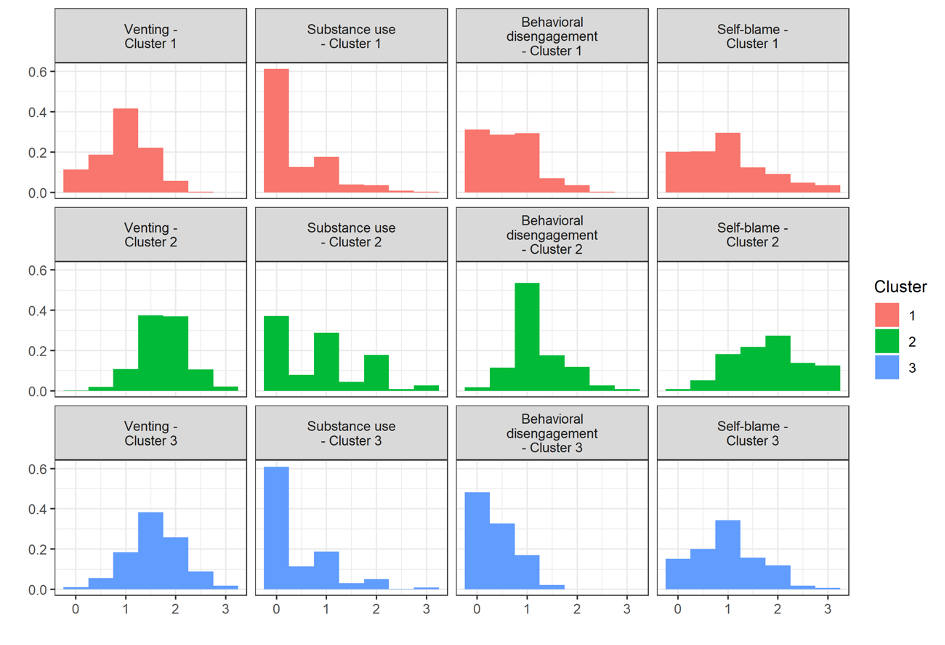


Histograms of the GHQ-28 and the IES-R scores at distinct clusters in medical professionals.


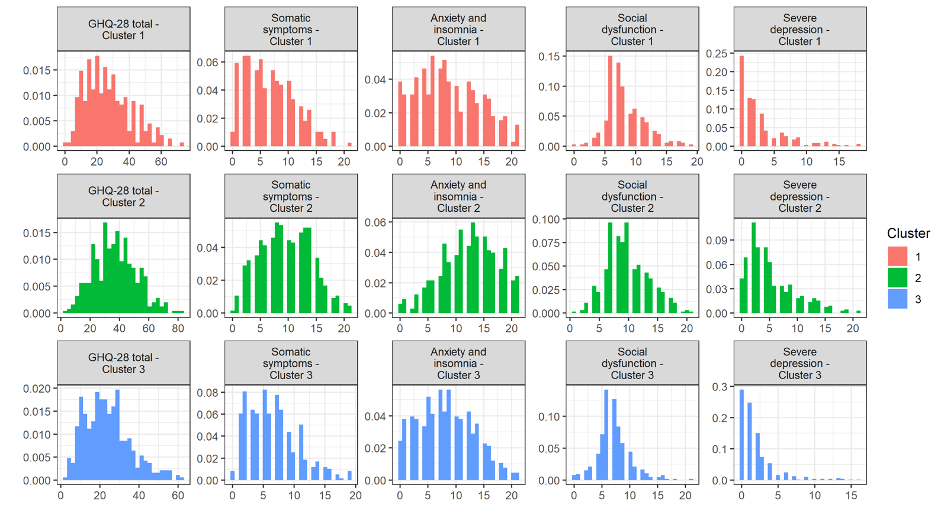


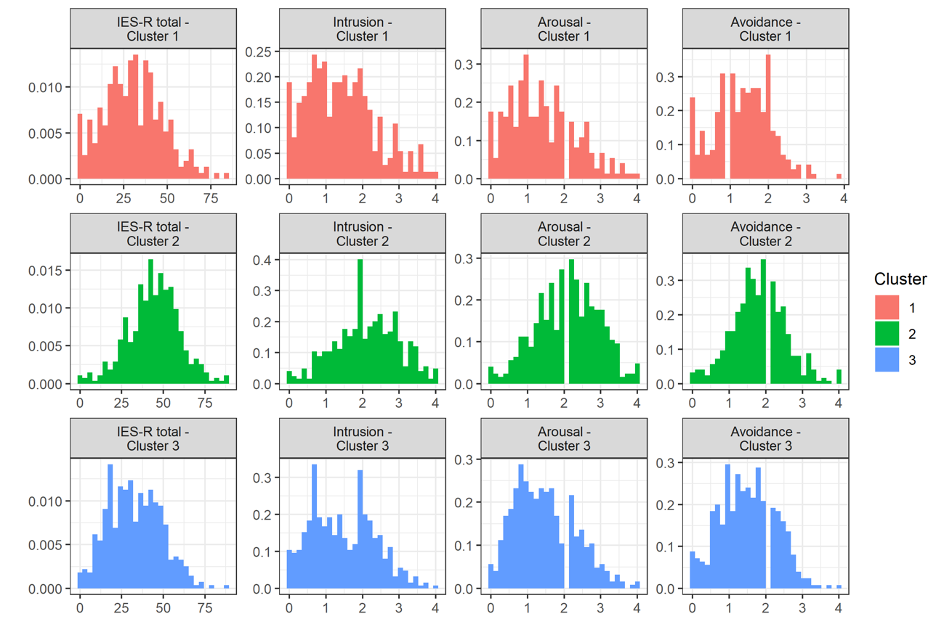


Histograms of the GHQ-28 and the IES-R scores at distinct clusters in non-medical professionals.


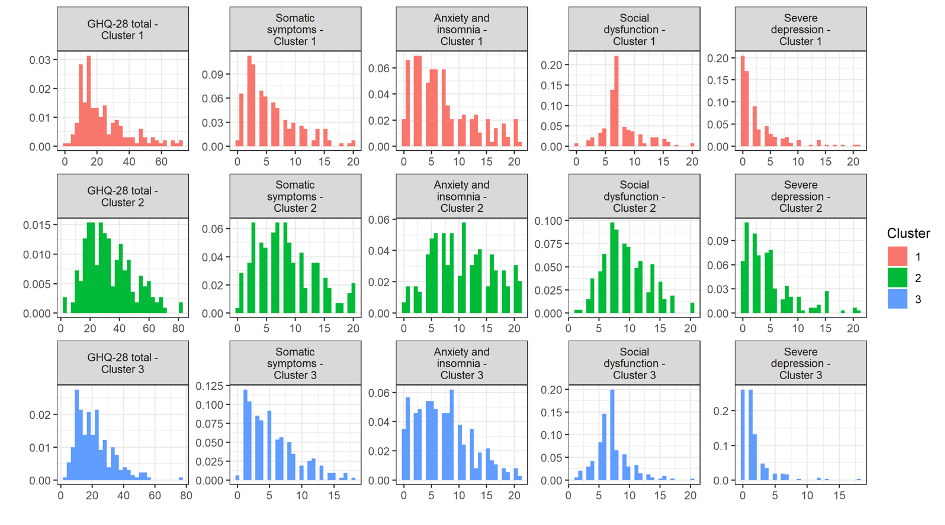


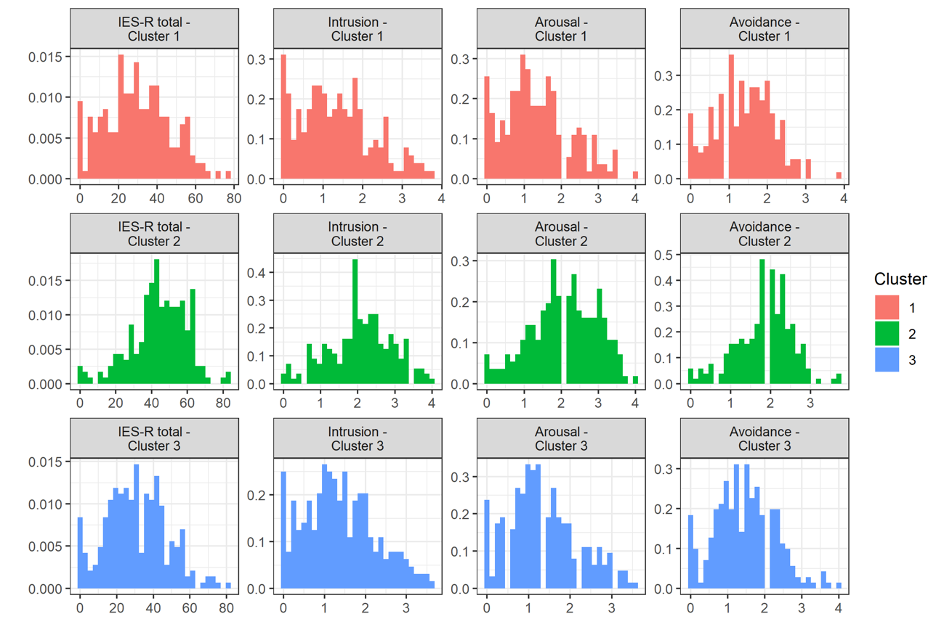

Supplement: Supplementary file 1 [file Data_Sheet_1.docx]
